# Supplementary material for: Evolutionary Dynamics and Functional Differences in Clinically Relevant Pen β‑Lactamases from Burkholderia spp
Source: J Chem Inf Model. 2025 May 2;65(10):5086–98. doi: 10.1021/acs.jcim.5c00271 (PMC12117567; doi:10.1021/acs.jcim.5c00271)
Supplement: Supplementary file 1 [file ci5c00271_si_001.pdf]

Supplementary Information

Evolutionary Dynamics and Functional Differences in Clinically relevant Pen β-Lactamases from *Burkholderia* spp.

Jing Gu,[1] Pratul K Agarwal,[2] Robert A Bonomo,[3,4,5,6,7,8] Shozeb Haider [1,9,10]

[1] UCL School of Pharmacy, University College London, London WC1N 1AX, U.K

[2] High-Performance Computing Center, Oklahoma State University, Stillwater, OK, USA

[3] Research Service, Louis Stokes Cleveland Department of Veterans Affairs Medical Center, Cleveland, OH, USA

[4] Department of Molecular Biology and Microbiology, Case Western Reserve University School of Medicine, Cleveland, OH, USA

[5] Department of Medicine, Case Western Reserve University School of Medicine, Cleveland, OH, USA

[6] Clinician Scientist Investigator, Louis Stokes Cleveland Department of Veterans Affairs Medical Center, Cleveland, OH, USA

[7] Departments of Pharmacology, Biochemistry, and Proteomics and Bioinformatics, Case Western Reserve University School of Medicine, Cleveland, OH, USA

[8] CWRU-Cleveland VAMC Center for Antimicrobial Resistance and Epidemiology (Case VA CARES) Cleveland, OH, USA

[9] University of Tabuk (PFSCBR), Tabuk, Saudi Arabia

[10] UCL Center for Advanced Research Computing, University College London, WC1H 9RL, U.K.

Corresponding author and email address:

Shozeb Haider (Shozeb.haider@ucl.ac.uk)

ORCID: Shozeb Haider: 0000-0003-2650-2925

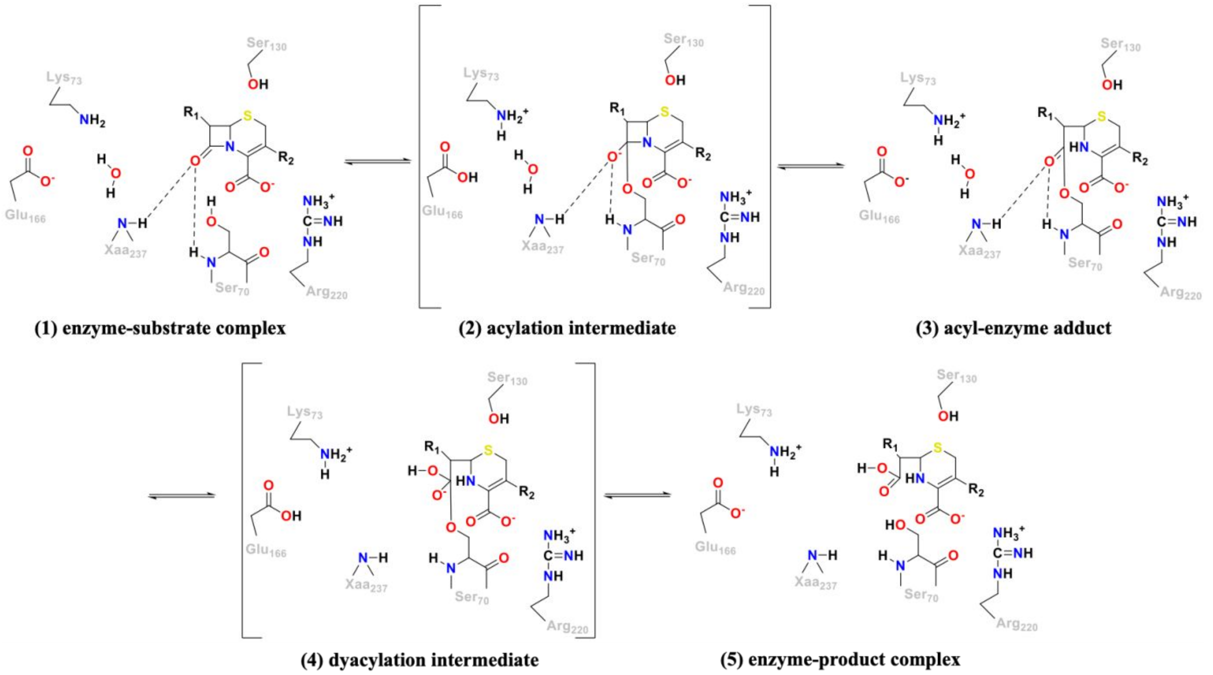

Figure S1. The proposed catalytic mechanism of Pen β-lactamases illustrated on the core structure of cephalosporin. The enzyme positions the β-lactam substrate in the active site by anchoring the carbonyl into the oxyanion hole formed by the backbone N-H of Ser70 and Xaa237 and stabilizing the carboxylate via a salt bridge to Arg220 (1). It activates Ser70 through deprotonation, enabling the nucleophilic attack on the β-lactam carbonyl to form the acylation intermediate (2). Acyl-enzyme adduct is formed with cleavage of the β-lactam ring (3). The enzyme then facilitates a proton transfer and activates a water molecule to attack the acyl-enzyme bond, which results in the form of diacylation intermediate (4). Finally, the enzyme is regenerated, and the inactivated β-lactam product is released from the enzyme-product complex (5) [1].

Table S1. k_{cat} values of Pen β-lactamases (s^{-1}). AMP: Ampicillin. AMX: amoxicillin. THIN: Cephalothin. CTX: Cefotaxime. NFN: Nitrocefin. IMI: Imipenem. ND: no measurable hydrolysis detected. N/A: Value not available in the literature.

	**AMP**	**AMX**	**THIN**	**CTX**	**NFN**	**IMI**
**PenA**	285±29 [1]	N/A	221±21 [2]	142±14 [1]	460±46 [2]	0.38±0.04
**PenI**	0.69±0.07 [1]	N/A	4.2±0.4 [1]	9.0±0.9 [1]	4.9±0.4 [1]	ND
**PenL**	N/A	83.5±10.8 [3]	N/A	1507±255.5 [3]	325±44 [3]	N/A
**PenP**	N/A	N/A	N/A	N/A	1088 [4]	N/A

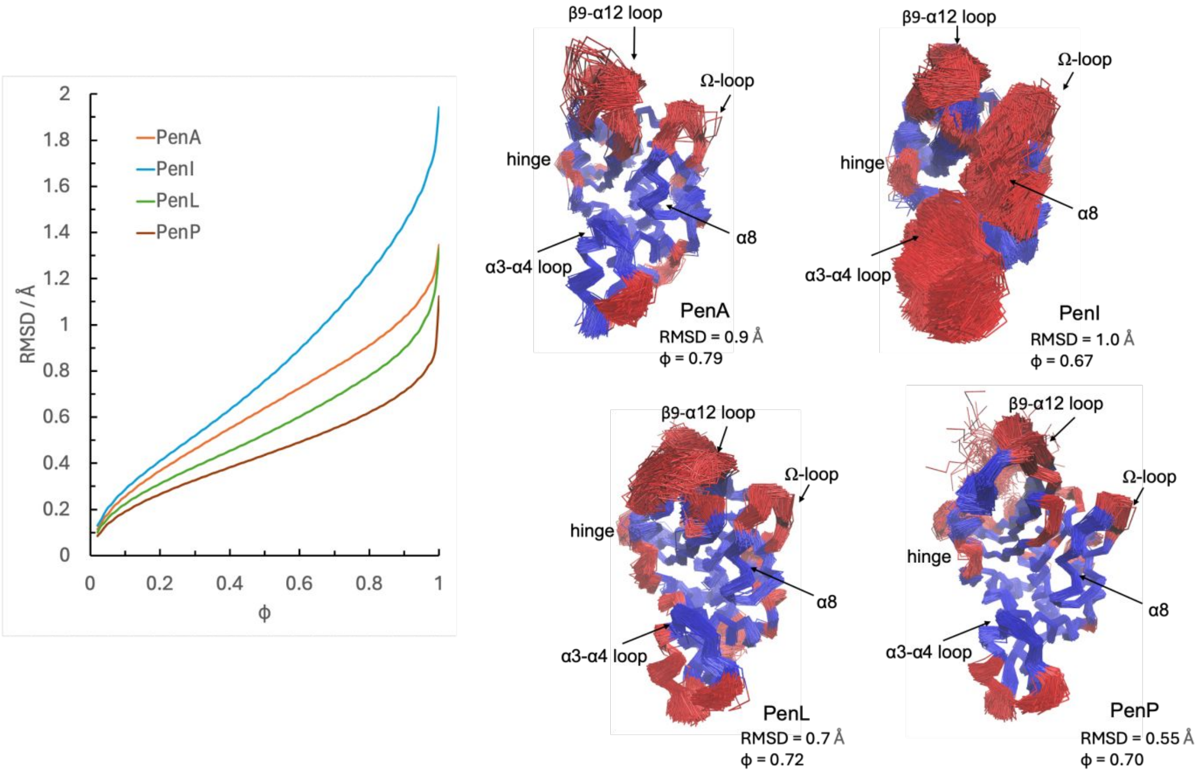

Figure S2. Core Cα RMSD superimposition from Pen simulations. The structural alignment was calculated from all trajectories with stride 24 and rendered to illustrate 745 uniformly separated frames. The least mobile Cα atoms are coloured blue and the most mobile atoms (red) provide the structural basis for the differential RMSDs.

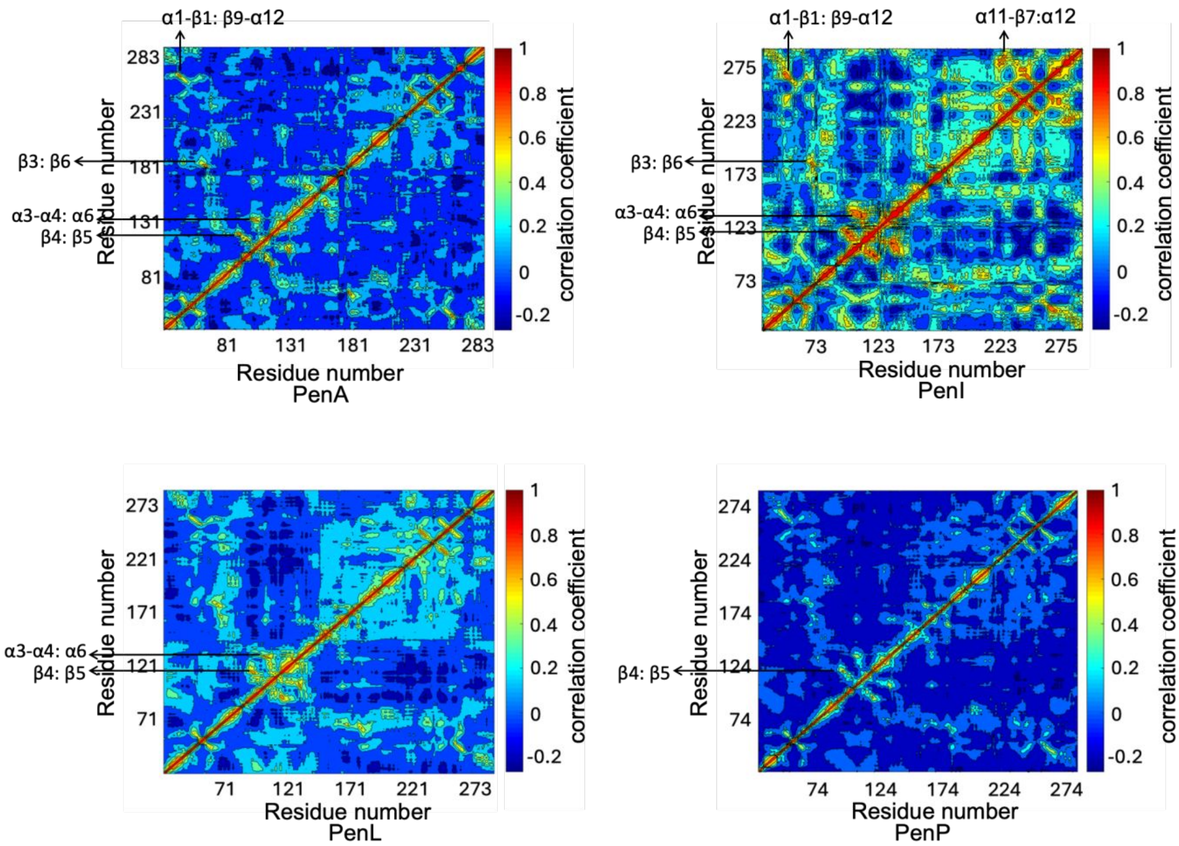

Figure S3. Dynamic cross-correlation map (DCCM) computed from Pen simulations. The regions showing significant positive correlations are highlighted.

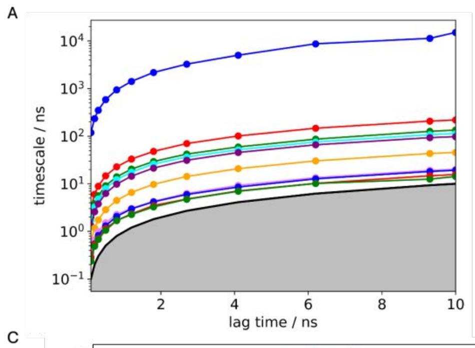

B

MFPT / ns:

	1	2	3	4	5	6	7
**1**	0.00	579.19	71414.38	89.75	95.35	241.24	285.01
**2**	1219.03	0.00	71420.15	103.24	101.39	237.78	264.46
**3**	5421.86	4802.14	0.00	4190.99	4052.72	4281.65	4349.59
**4**	1300.52	678.67	71373.69	0.00	59.08	208.70	240.25
**5**	1332.52	714.60	71312.68	102.54	0.00	188.37	259.90
**6**	1424.97	799.75	71456.32	202.08	137.46	0.00	161.89
**7**	1441.23	805.76	71497.18	206.25	181.06	134.11	0.00

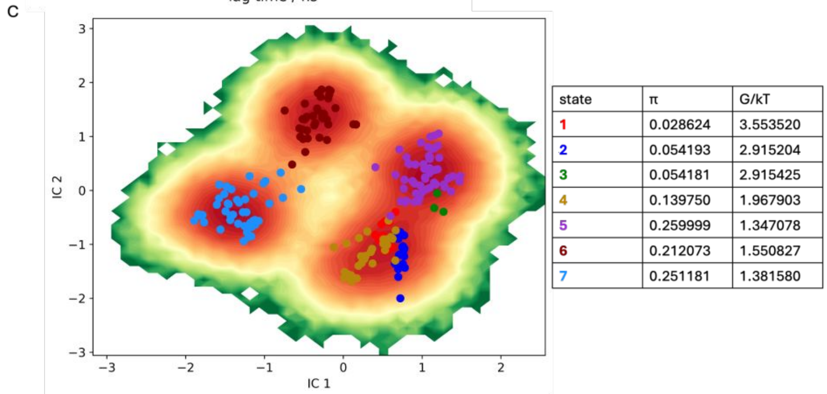

state	π	G/kT
1	0.028624	3.553520
2	0.054193	2.915204
3	0.054181	2.915425
4	0.139750	1.967903
5	0.259999	1.347078
6	0.212073	1.550827
7	0.251181	1.381580

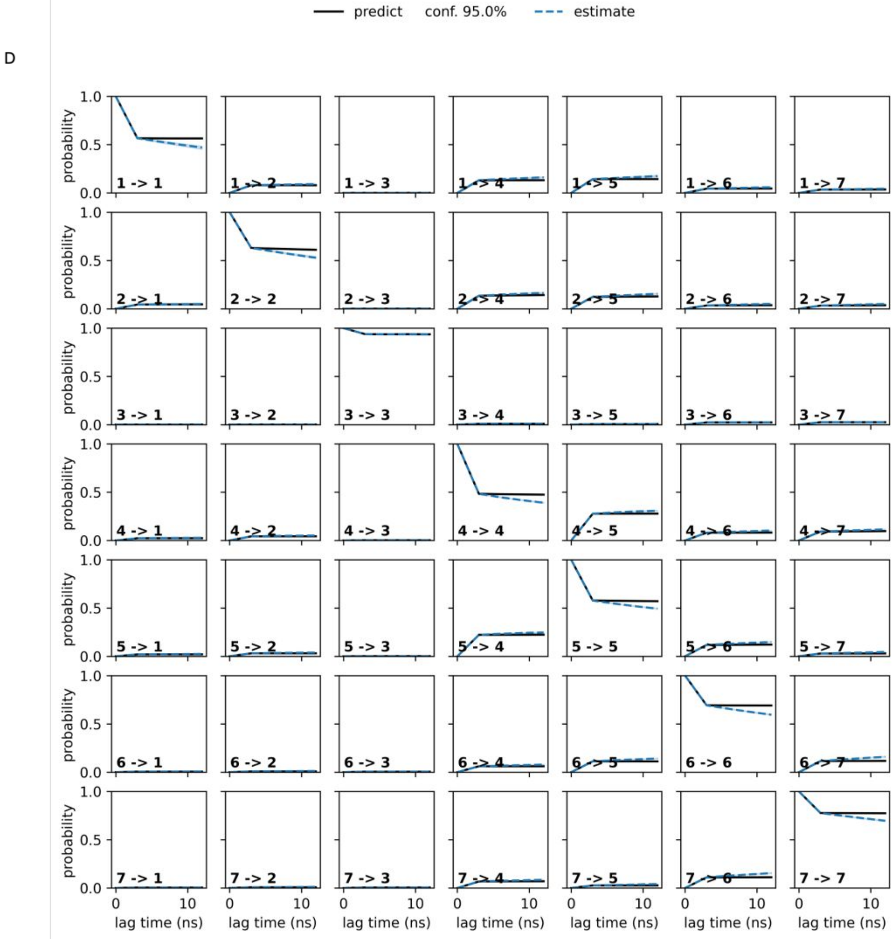

Figure S4. PenA Markov State Model. A. Implied timescales (ITS) plot. B. Mean first passage times between metastable states per ns. C. The distribution of cluster centres highlighting the presence of the metastable states. D. Chapman-Kolmogorov (CK) test plots

A

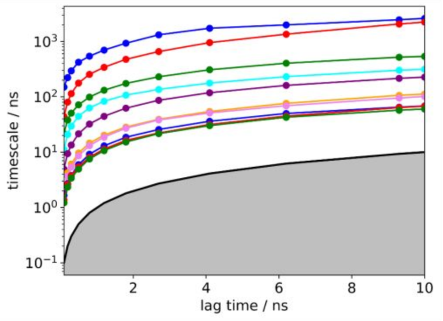

B

MFPT / ns:

	1	2	3	4	5	6	7	8
1	0.00	7552.79	45.67	2764.04	2577.86	4754.24	1409.00	2220.82
2	26948.05	0.00	21035.75	88.22	365.56	2092.76	1061.16	1607.25
3	3962.67	7475.00	0.00	2684.60	2498.28	4674.79	1328.87	2140.77
4	27133.66	4849.69	21200.10	0.00	191.45	1719.07	925.55	1466.91
5	27040.18	5316.82	21104.61	219.40	0.00	2183.84	796.88	1335.42
6	27538.62	5434.16	21604.38	251.72	587.38	0.00	1317.67	1855.99
7	26330.58	6313.84	20388.77	1233.38	1013.92	3217.79	0.00	501.87
8	26643.68	6594.54	20702.22	1513.39	1293.35	3494.36	208.28	0.00

C

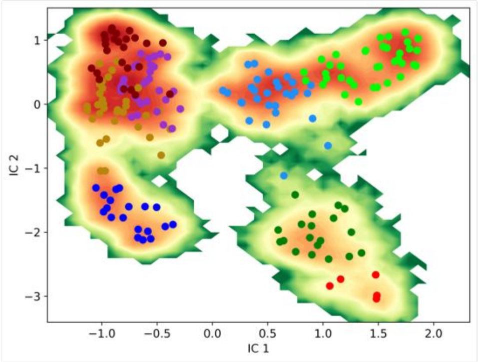

state	π	G/kT
1	0.013463	4.307812
2	0.026853	3.617385
3	0.051612	2.964000
4	0.116686	2.148266
5	0.135323	2.000088
6	0.145435	1.928024
7	0.213052	1.546221
8	0.297576	1.212087

D

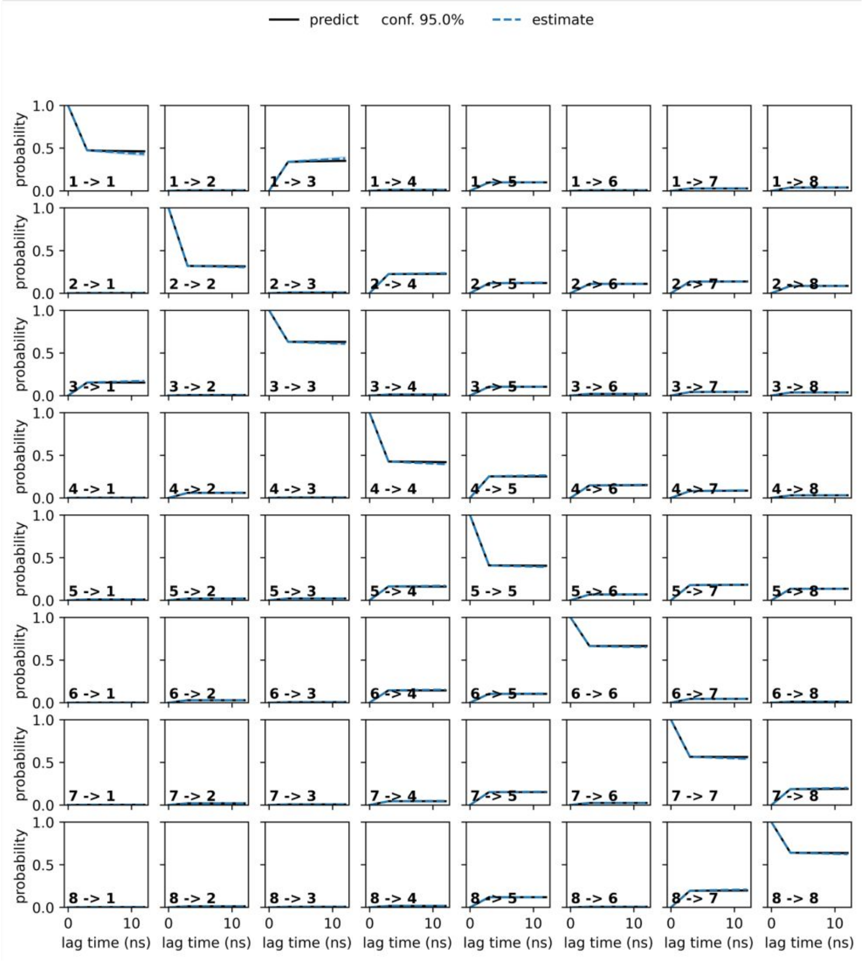

Figure S5. PenI Markov State Model. A. Implied timescales (ITS) plot. B. Mean first passage times between metastable states per ns. C. The distribution of cluster centres highlighting the presence of the metastable states. D. Chapman-Kolmogorov (CK) test plots

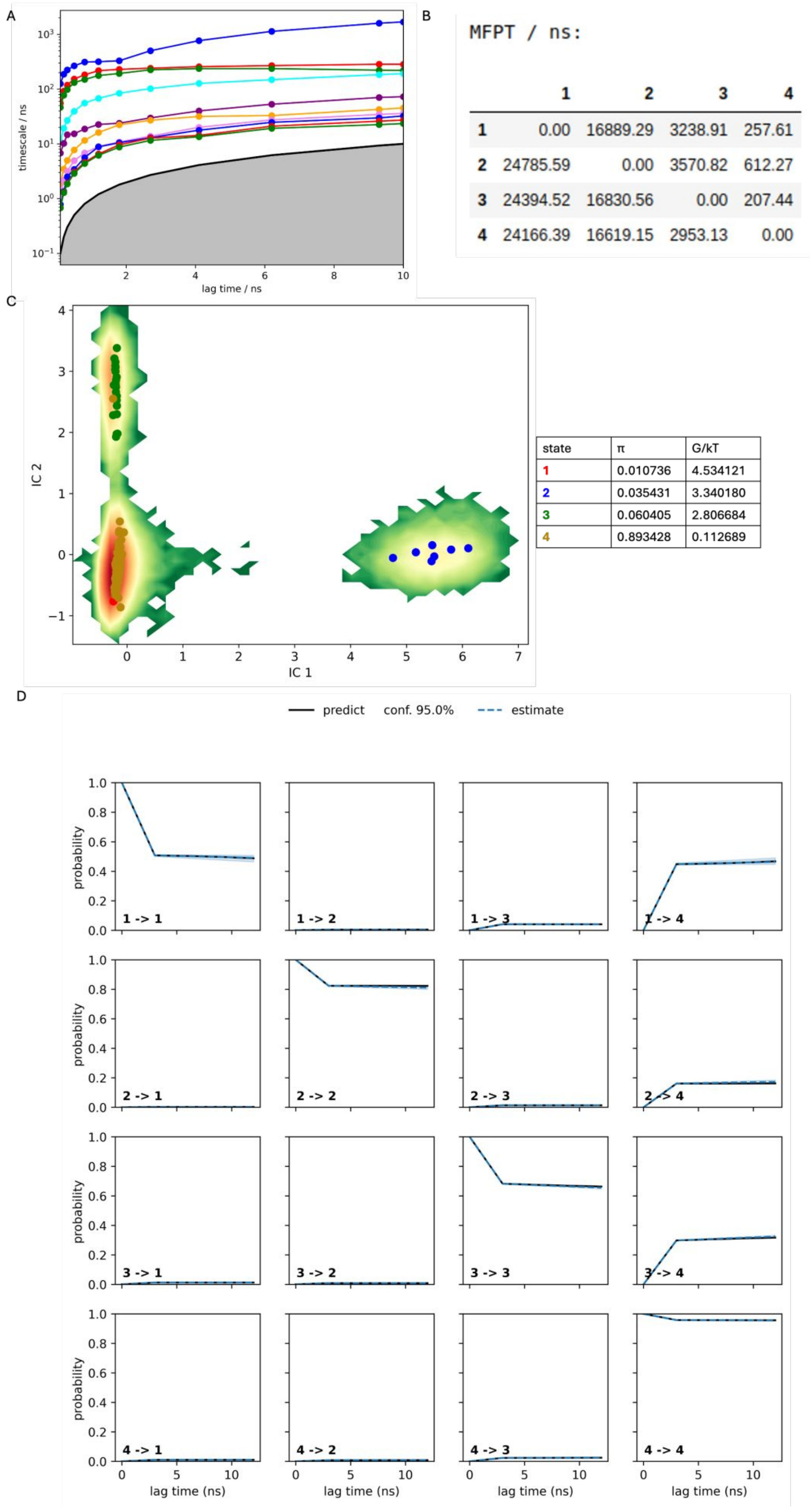

MFPT / ns:

	1	2	3	4
1	0.00	16889.29	3238.91	257.61
2	24785.59	0.00	3570.82	612.27
3	24394.52	16830.56	0.00	207.44
4	24166.39	16619.15	2953.13	0.00

state	π	G/kT
1	0.010736	4.534121
2	0.035431	3.340180
3	0.060405	2.806684
4	0.893428	0.112689

Figure S6. PenL Markov State Model. A. Implied timescales (ITS) plot. B. Mean first passage times between metastable states per ns. C. The distribution of cluster centres highlighting the presence of the metastable states. D. Chapman-Kolmogorov (CK) test plots

A

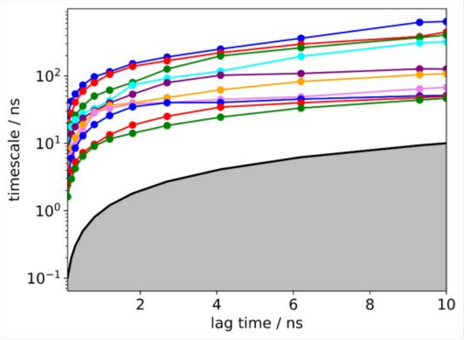

B

MFPT / ns:

	1	2	3	4	5
**1**	0.00	6298.68	4988.17	68.20	305.93
**2**	37130.88	0.00	4835.88	1848.35	97.71
**3**	37222.02	6233.94	0.00	1945.99	185.63
**4**	34301.27	6164.66	4855.66	0.00	173.39
**5**	37005.54	5994.96	4664.74	1725.52	0.00

C

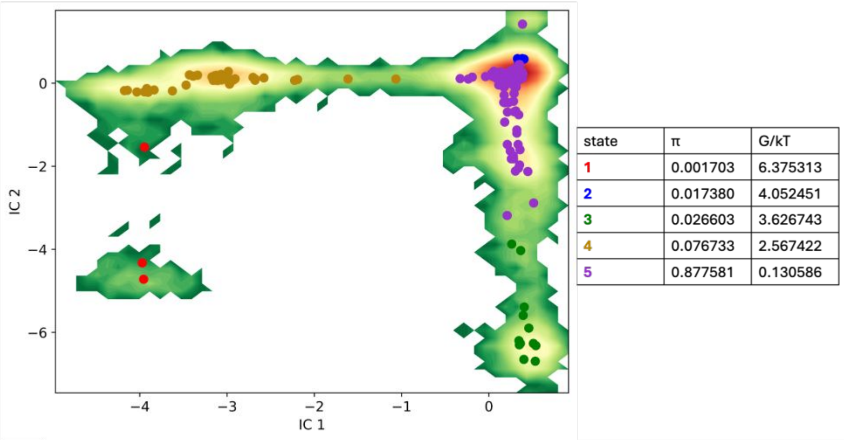

state	π	G/kT
1	0.001703	6.375313
2	0.017380	4.052451
3	0.026603	3.626743
4	0.076733	2.567422
5	0.877581	0.130586

D

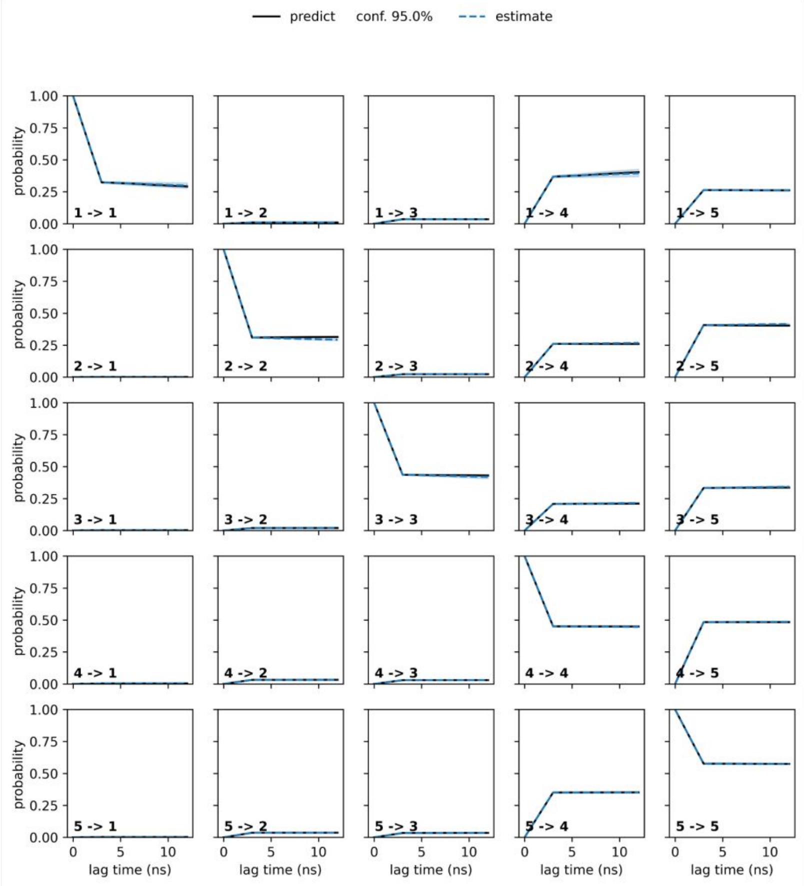

Figure S7. PenP Markov State Model. A. Implied timescales (ITS) plot. B. Mean first passage times between metastable states per ns. C. The distribution of cluster centres highlighting the presence of the metastable states. D. Chapman-Kolmogorov (CK) test plots

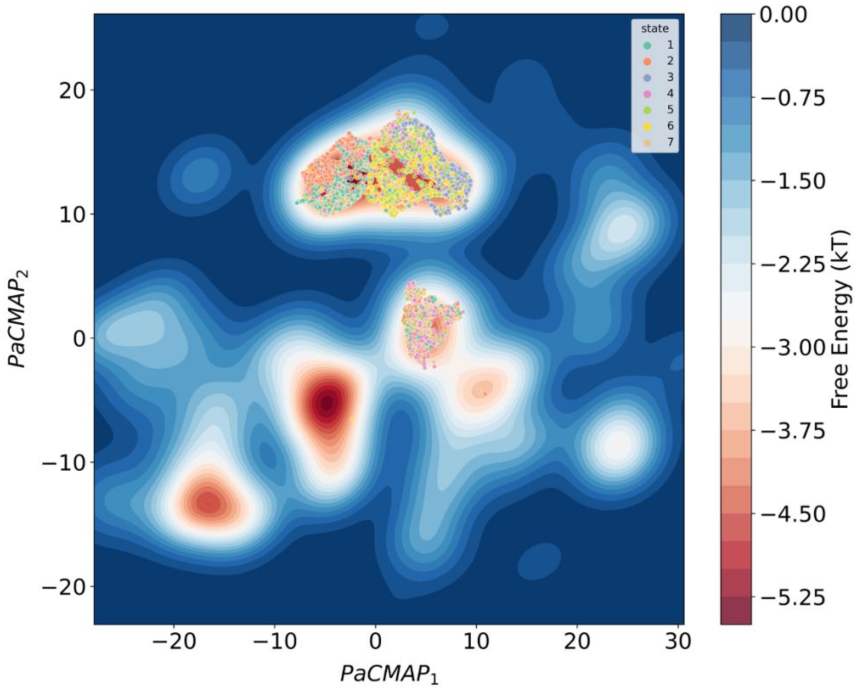
state
1
2
3
4
5
6
7
0.00
−0.75
−1.50
−2.25
−3.00
−3.75
−4.50
−5.25
Free Energy (kT)
20
10
0
−10
−20
−20
−10
0
10
20
30
$PaCMAP_2$
$PaCMAP_1$

PenA

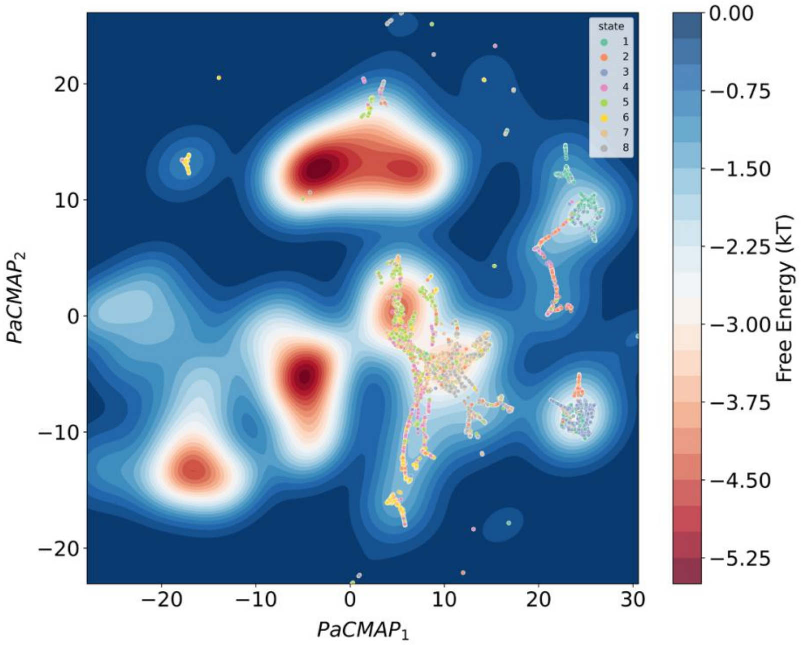
state
1
2
3
4
5
6
7
8
0.00
−0.75
−1.50
−2.25
−3.00
−3.75
−4.50
−5.25
Free Energy (kT)
20
10
0
−10
−20
−20
−10
0
10
20
30
$PaCMAP_2$
$PaCMAP_1$

PenI

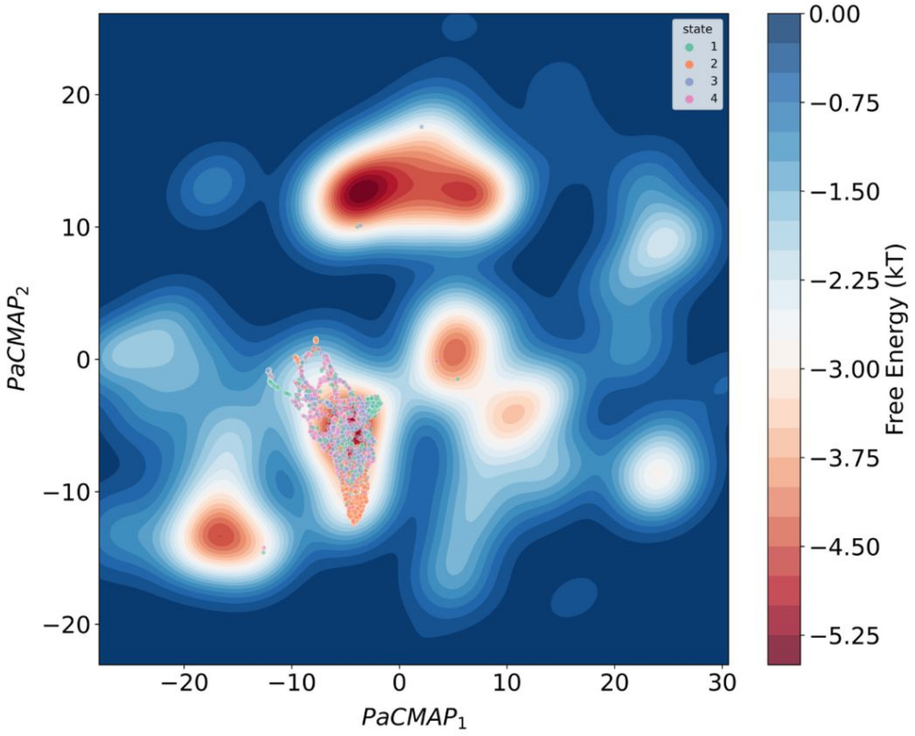

PenL

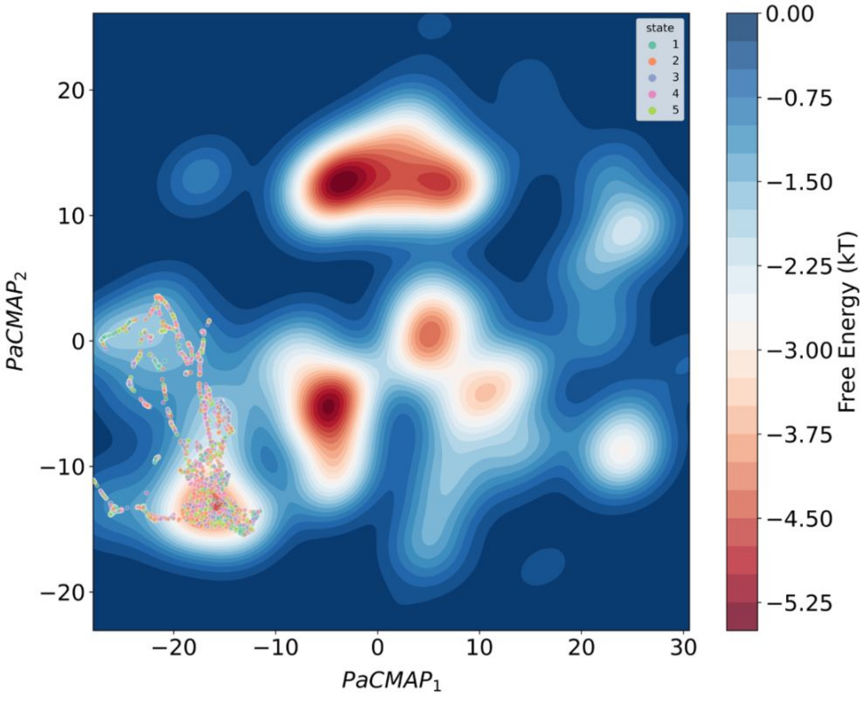

PenP

Figure S8. Convolutional variational autoencoder (CVAE)-based deep learning analysis.

References

(1) Papp-Wallace, K. M.; Taracila, M. A.; Gatta, J. A.; Ohuchi, N.; Bonomo, R. A.; Nukaga, M. Insights into beta-lactamases from Burkholderia species, two phylogenetically related yet distinct resistance determinants. *J Biol Chem* **2013**, *288* (26), 19090-19102. DOI: 10.1074/jbc.M113.458315.

(2) Becka, S. A.; Zeiser, E. T.; Barnes, M. D.; Taracila, M. A.; Nguyen, K.; Singh, I.; Sutton, G. G.; LiPuma, J. J.; Fouts, D. E.; Papp-Wallace, K. M. Characterization of the AmpC beta-Lactamase from Burkholderia multivorans. *Antimicrob Agents Chemother* **2018**, *62* (10). DOI: 10.1128/AAC.01140-18.

(3) Yi, H.; Choi, J. M.; Hwang, J.; Prati, F.; Cao, T. P.; Lee, S. H.; Kim, H. S. High adaptability of the omega loop underlies the substrate-spectrum-extension evolution of a class A beta-lactamase, PenL. *Sci Rep* **2016**, *6*, 36527. DOI: 10.1038/srep36527.

(4) Au, H.-W.; Tsang, M.-W.; So, P.-K.; Wong, K.-Y.; Leung, Y.-C. Thermostable β-Lactamase Mutant with Its Active Site Conjugated with Fluorescein for Efficient β-Lactam Antibiotic Detection. *ACS Omega* **2019**, *4* (24), 20493-20502. DOI: 10.1021/acsomega.9b02211.
